# A Tale of Two Evils: Iatrogenic Propagation of Spontaneous Coronary Artery Dissection

**DOI:** 10.7759/cureus.45569

**Published:** 2023-09-19

**Authors:** Juan Enciso, Madeline Zipperer, Luke Casals, Daniela R Crousillat

**Affiliations:** 1 Department of Internal Medicine, University of South Florida Morsani College of Medicine, Tampa, USA; 2 Heart and Vascular Institute, Tampa General Hospital, Tampa, USA; 3 Department of Cardiovascular Sciences, University of South Florida Morsani College of Medicine, Tampa, USA

**Keywords:** spontaneous coronary artery dissection, recurrent spontaneous coronary artery dissection, multivessel scad, scad management, scad and fmd, scad

## Abstract

Spontaneous coronary artery dissection (SCAD) is a non-traumatic and non-iatrogenic intimal separation of the coronary arterial wall. While poorly understood, its mechanism confers higher prevalence in younger females, and it is responsible for 25% of acute coronary syndromes (ACS) in women under 50 years of age. SCAD is primarily diagnosed via coronary angiography; however, intraluminal contrast injection and percutaneous coronary interventions (PCI) are associated with an increased risk of propagation and extension of the dissection leading to increased risk of morbidity and mortality. We present the case of a 48-year-old female with multivessel SCAD and subsequent iatrogenic dissection following contrast injection requiring multiple PCI for medical treatment of refractory cardiac angina.

## Introduction

Spontaneous coronary artery dissection (SCAD) presents as a sudden separation of the intramedial coronary arterial wall due to the formation of an intramural hematoma, often followed by subsequent intimal tear. The mean average age of presentation is estimated between 52 ± 9 years of age, with 62% of these being postmenopausal women [1,2]. SCAD is also more prevalent among women with a unique substrate for presentation, including hormonal fluctuations, with an increased incidence in the post-partum period [1,2]. Other predisposing factors commonly associated with SCAD include systemic inflammatory diseases, arteriopathies such as fibromuscular dysplasia (FMD), connective tissue disorders such as Marfan’s or Ehlers-Danlos syndromes, and significant emotional or physical stressors [2,3]. While the gold standard for diagnosing SCAD is optical coherence tomography (OCT) or intravascular ultrasound (IVUS), SCAD is often underdiagnosed due to varying angiographic presentations distinct from typical atherosclerotic coronary disease. Additionally, diagnostic angiography, intravascular imaging or manipulation, and percutaneous coronary interventions (PCI) are associated with an increased risk of dissection propagation due to the vulnerability of the vessel wall.

## Case presentation

**Figure 1:**
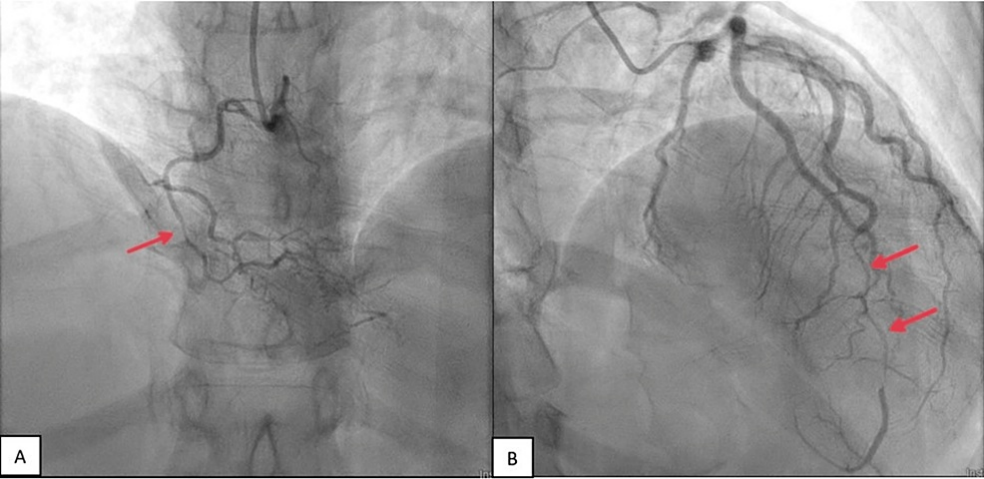
(A) Initial coronary angiogram showing RCA with diffuse luminal narrowing with the presence of dissection of the mid to distal RCA. (B) Coronary angiogram demonstrating a long tapering distal LAD dissection with partial reconstitution of vessel size and flow distal to lesion prior to initial LAD PCI

A 48-year-old G2P2 woman with a medical history of hypertension, insulin-dependent diabetes, systemic lupus erythematosus (SLE), and a remote history of recurrent angina initially presented to an outside institution for recurrent substernal chest pain with associated dyspnea. On presentation, ECG was non-diagnostic with nonspecific ST changes, high sensitivity troponin rose from 26 ng/L on admission to 277 ng/mL (normal range ≤ 14 ng/L), and echocardiogram demonstrated mildly depressed left ventricular ejection fraction of 45-50% with apical hypokinesis. Coronary angiography revealed multivessel disease involving the mid-right coronary artery (RCA) and long, diffuse narrowing of the mid-left anterior descending artery (LAD) (Figure 1A-1B), which was thought to be the culprit lesion. The patient underwent PCI with the placement of two drug-eluting stents (DES) to the mid-LAD without complications. She was discharged in stable condition on dual antiplatelet therapy (DAPT).

**Figure 2:**
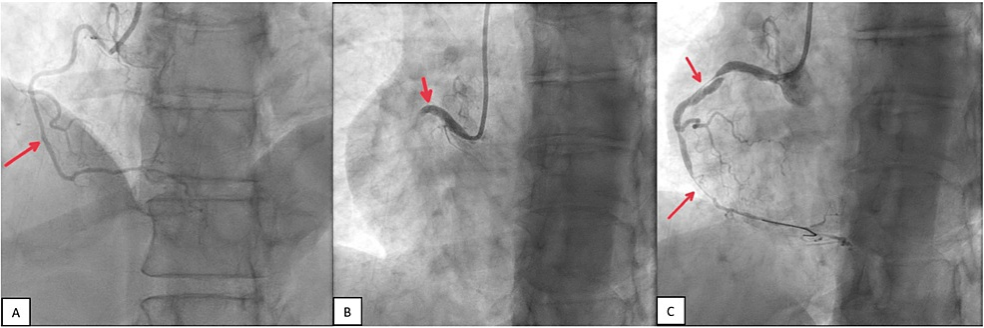
(A) Second coronary angiogram showing a persistent reduction in vessel caliber but relative improvement in distal RCA caliber and flow. (B) Subsequent re-look injection into RCA leading to proximal RCA dissection. (C) Retrograde type I dissection with evidence of "intimal" flap extending to proximal RCA with near complete obstruction of flow distally

Unfortunately, she re-presented two weeks post-LAD PCI due to persistent angina and progressive dyspnea. Repeat coronary angiography demonstrated patent LAD stents and initial relative improvement in distal RCA caliber and flow (compared to the previous study two weeks prior). However, repeat injection into the RCA led to a proximal RCA dissection requiring three overlapping DES (Figure 2A-2B). The patient's symptoms resolved within 24-48 hours, and she was discharged in stable condition on appropriate medical therapy.

**Figure 3:**
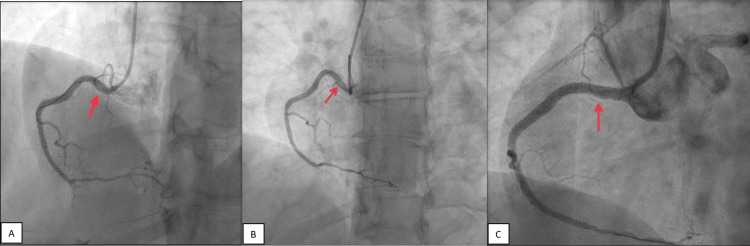
(A) Last coronary angiogram from second hospitalization post-PCI. (B) Coronary angiogram at our institution (third hospitalization) showing a new proximal dissection from the previously placed mid-to-distal RCA stents to the right coronary ostium. (C) Coronary angiogram with the placement of 1x DES from the mid-RCA to the RCA ostium

One day after discharge, she presented to our institution for recurrent chest pain and laboratory evidence of cardiac ischemia (troponin I 0.472 ng/mL, normal range < 0.04 ng/mL). Re-look coronary angiogram demonstrated patent LAD and RCA stents with a new proximal dissection from the previously placed mid-to-distal RCA stents to the right coronary ostium (Figure 3A-3B). A comprehensive review of her coronary angiography films from recent hospitalizations demonstrated initial presentation with unrecognized multivessel SCAD with type 2 lesions involving the distal LAD and mid-distal RCA, followed by iatrogenic antegrade RCA dissection from the contrast injection during the second angiogram. It was determined that the primary etiology of refractory angina was due to multivessel SCAD and was unfortunately exacerbated by repeated diagnostic coronary angiograms and coronary interventions. Unfortunately, due to persistent and refractory symptoms, she underwent additional placement of 1 DES from the mid-RCA to the RCA ostium with marked symptom improvement. CT angiogram of the head, neck, abdomen, and pelvis demonstrated no evidence of underlying FMD or arteriopathy. She was discharged on DAPT and beta-blocker therapy with a referral to cardiac rehabilitation.

## Discussion

Early epidemiological data suggests that SCAD is the cause of 4% of all acute coronary syndrome (ACS)-reported cases. However, its under-recognition likely underestimated the true incidence of SCAD [4,5]. While SCAD contributes to almost 25% of all ACS presentations in women younger than 50, it is now recognized as an essential cause of ACS in older populations [6]. In the Canadian SCAD cohort study, one of the largest multicenter SCAD registries to date, 88.5% of individuals were female, with a mean age of presentation of 51.8 years and 9.2% older than 65 years of age [3]. While SCAD's pathophysiology remains poorly understood, predisposing conditions associated with SCAD include arteriopathies, most notably FMD, psychological and physical stressors, and hormonal influences, including pregnancy, post-partum, and menopause [7]. Initial reports suggested a link between SCAD and autoimmune diseases, including SLE, such as in our patient; however, recent case-control studies have not identified a strong association [8]. The most consistent predisposing factor is FMD, with over 70% of patients with confirmed SCAD demonstrating a concurrent diagnosis of FMD during the subsequent screening performed with angiography or computed tomographic angiography/MR angiography [2].

The clinical presentation of SCAD can range from classic anginal symptoms to less common isolated non-cardiac prodromes [3]. ST-elevation myocardial infarction (STEMI) presentation was evident in 25% of patients, while non-ST-elevation myocardial infarction (NSTEMI) represented the remainder. Less than 1% had no elevated cardiac biomarkers [1]. Prospective, multicenter registry data demonstrates that the LAD is the most frequently affected vessel for NSTEMI presentations, while left main (LM) disease is more commonly involved among STEMI presentations [1]. Lastly, single vessel involvement is the most common presentation, with only 10% of patients having multivessel involvement [1,9]. The diagnostic gold standard for SCAD is based on OCT or IVUS with three main subtype classifications: Type 1: presence of multiple radiolucent lumens or contrast wall staining, Type 2: diffuse narrowing with either normal adjacent segments or narrowing that extends to the vessel tip, and Type 3: atherosclerotic mimicker with focal or tubular stenosis [10].

Type 2 is the most common SCAD subtype, which can often be missed and mistaken for diffuse narrowing or a small caliber vessel, given the absence of the classic angiographic finding of dissection with multiple lumens [1]. In this case, Type 2 SCAD was thought to be the initial etiology of her sentinel presentation. Additional intracoronary imaging, such as OCT and IVUS, can improve diagnostic accuracy; however, it must be weighed against the risk of further propagation of SCAD. The utility of cardiac computed tomography angiography for suspected SCAD is under investigation as an attractive, less invasive diagnostic option without the risks of dissection propagation, which can be associated with routine contrast injections during diagnostic coronary angiography; however, coronary angiography with intracoronary imaging remains the gold standard in the context of acute presentation with ACS [11-13]. Coronary angiography and PCI in SCAD are associated with an increased risk of entry into the false lumen, an extension of the intramural hematoma with balloon dilation, and stent malposition leading to an increased risk of intraluminal thrombosis and should, therefore, be performed meticulously and only when clinically indicated [2,14]. Conservative medical management is preferred for stable patients without hemodynamic compromise. In cases associated with refractory angina/ischemia, cardiogenic shock, sustained ventricular arrhythmias, or LM involvement, PCI may be performed with consideration of coronary artery bypass graft for patients with LM dissection or >2 vessel proximal SCAD lesions. It is essential to highlight that conservative management is favored as most SCAD lesions display intraluminal healing and repair within six weeks post-diagnosis.

## Conclusions

This case highlights the importance of diagnosing SCAD, particularly among young women presenting with ACS. Suspicion for SCAD should be heightened based on age, sex, clinical presentation, and precipitating factors. It is essential to accurately identify the presence of SCAD to ensure appropriate treatment, as it varies from the traditional management of atherosclerotic coronary disease. Conservative medical management is preferred in stable patients diagnosed with SCAD, as most lesions angiographically heal without intervention, and PCI is typically associated with added complications and low success rates.

This patient underwent multiple diagnostic coronary angiograms and PCIs, resulting in the extension and progression of existent dissections highlighting the unique risks associated with SCAD. Early identification and consideration of SCAD as a potential diagnosis for ACS can help tailor the treatment strategy and potentially prevent downstream complications, as highlighted by this case.
